# Thenar Muscle Motor Imagery Increases Spinal Motor Neuron Excitability of the Abductor Digiti Minimi Muscle

**DOI:** 10.3389/fnhum.2021.753200

**Published:** 2021-12-02

**Authors:** Yoshibumi Bunno, Toshiaki Suzuki

**Affiliations:** Graduate School of Health Sciences, Graduate School of Kansai University of Health Sciences, Osaka, Japan

**Keywords:** motor imagery (MI), F-wave, spinal motor neuron (SMN), enslaving effect, abductor digiti minimi (ADM)

## Abstract

When a person attempts intended finger movements, unintended finger movement also occur, a phenomenon called “enslaving”. Given that motor imagery (MI) and motor execution (ME) share a common neural foundation, we hypothesized that the enslaving effect on the spinal motor neuron excitability occurs during MI. To investigate this hypothesis, electromyography (EMG) and F-wave analysis were conducted in 11 healthy male volunteers. Initially, the EMG activity of the left abductor digiti minimi (ADM) muscle during isometric opposition pinch movement by the left thumb and index finger at 50% maximal effort was compared with EMG activity during the Rest condition. Next, the F-wave and background EMG recordings were performed under the Rest condition, followed by the MI condition. Specifically, in the Rest condition, subjects maintained relaxation. In the MI condition, they imagined isometric left thenar muscle activity at 50% maximal voluntary contraction (MVC). During ME, ADM muscle activity was confirmed. During the MI condition, both F-wave persistence and the F-wave/M-wave amplitude ratio obtained from the ADM muscle were significantly increased compared with that obtained during the Rest condition. No difference was observed in the background EMG between the Rest and MI conditions. These results suggest that MI of isometric intended finger muscle activity at 50% MVC facilitates spinal motor neuron excitability corresponding to unintended finger muscle. Furthermore, MI may induce similar modulation of spinal motor neuron excitability as actual movement.

## Introduction

Motor imagery (MI) is defined as a dynamic mental state that reproduces a specific motor action within working memory without any overt movement (Decety and Grèzes, 1999; Guillot et al., 2012). MI allows patients with difficulty in volitional movements, such as post-stroke patients, to mentally practice a target motor task. Indeed, MI has been applied to physical therapy for disorders of the central nervous system (Jackson et al., 2001). Numerous neurophysiological studies using positron emission tomography, functional magnetic resonance imaging, and transcranial magnetic stimulation (TMS) have provided valid evidence that MI shares many neural networks with motor execution (ME) (Mizuguchi et al., 2012; Hanakawa, 2016). At the spinal level, MI has been shown to increase the spinal motor neuron excitability (Taniguchi et al., 2008; Hara et al., 2010); however, other studies have reported that MI fails to alter spinal motor neuron excitability (Kasai et al., 1997; Hashimoto and Rothwell, 1999; Stinear et al., 2006). Taniguchi et al. (2008) and Hara et al. (2010) investigated the effect of MI on the spinal motor neuron excitability using F-waves, whereas Kasai et al. (1997) and Hashimoto and Rothwell (1999) used the Hoffmann reflex (H-reflex) to assess the spinal motor neuron excitability. There are differences in the mechanisms of occurrence between F-waves and H-reflex. F-waves are so named because they were initially recorded in small foot muscles (Magladery and McDougal, 1950). F-waves are compound action potentials resulting from the re-excitation of spinal anterior horn cells by an antidromic impulse following distal electrical stimulation of α-motor neurons (Kimura, 1974; Mesrati and Vecchierini, 2004; Fisher, 2007). Thus, F-waves may reflect changes in the spinal motor neuron excitability only. On the other hand, H-reflex reflects the response of the motor neuron pool to a volley from large-diameter primary muscle spindle afferents (McNeil et al., 2013), the presynaptic mechanism affects H-reflex amplitude. Additionally, previous studies by Hashimoto and Rothwell (1999) and Stinear et al. (2006) adopted phasic thumb tapping movements at a frequency of 1 Hz as the MI task. Previously, we investigated whether MI and action observation of cyclic thumb opposition movements at a frequency of 1 Hz affect spinal motor neuron excitability using F-waves (Bunno and Suzuki, 2020). As a result, combined action observation and MI failed to facilitate spinal motor neuron excitability significantly over that of action observation alone. This previous result suggested that MI of cyclic thumb opposition movements may have difficulty to increase spinal motor neuron excitability. Thus, the conflicting findings among these previous studies may be explained by methodological differences and further studies are required.

When humans attempt to perform intended individual finger movements, other fingers may also move. This involuntary movement by unintended fingers is called “enslaving” (Zatsiorsky et al., 1998, 2000). Thus, if MI has a similar neural foundation as ME, the enslaving effect may be observed in the other fingers during MI of a given finger movement. During hand-closing movement, the opponens pollicis (OP) and first dorsal interosseus (FDI) muscles are coactivated. For both the OP and FDI muscles, the amplitudes of motor evoked potentials (MEPs), which is evoked by applying TMS over the primary motor cortex and is an index of corticospinal excitability, were increased significantly while subjects imagining movement of the thumb toward the base of the little finger (Marconi et al., 2007). Furthermore, during MI of hand-closing or hand-opening movement, the amplitudes of MEPs of both agonist and antagonist muscles were significantly increased, similar to that observed in actual movement (Fadiga et al., 1999). Whereas these studies indicate that the enslaving effect occurs during MI, Rossini et al. (1999) and Facchini et al. (2002) failed to observe the enslaving effect during MI using TMS. Furthermore, those studies could not confirm the enslaving effect during MI even at the spinal level.

An isometric pinching movement is often used in holding small objects (e.g., buttons, coins, pens, and chopsticks) in daily living. Using F-wave analysis, our laboratory has shown that MI of isometric opposition pinch movement by the thumb and index finger at 50% maximal voluntary contraction (MVC) facilitates spinal motor neuron excitability corresponding to the abductor pollicis brevis (APB) muscle (Suzuki et al., 2013; Bunno et al., 2014, 2015; Bunno, 2018, 2019). As described above, F-waves are defined as compound action potentials resulting from the re-excitation (“backfiring”) of spinal anterior horn cells by an antidromic impulse following distal electrical stimulation of α-motor neurons (Kimura, 1974; Mesrati and Vecchierini, 2004; Fisher, 2007). Given that the amplitude of F-waves was increased when corticospinal descending volleys collide with antidromic peripheral volleys, F-waves are considered to be a reliable marker of spinal motor neuron excitability (Mercuri et al., 1996). However, no studies have addressed the relationship between MI of isometric opposition pinch movement by instructed fingers and spinal motor neuron excitability corresponding to uninstructed finger muscles. Thus, in this study, we aimed to investigate whether MI of an isometric opposition pinch movement by the thumb and index finger at 50% MVC alters spinal motor neuron excitability of the abductor digiti minimi (ADM) muscle using electromyography (EMG) and F-wave analysis.

## Materials and Methods

### Subjects

We aimed to clarify the enslaving effect on spinal motor neuron excitability corresponding to uninstructed finger muscles by applying MI. Thus, subjects with sufficient MI ability should be recruited to participate in this study. Because aging (Saimpont et al., 2013) and gender (Campos, 2014) affects MI ability, eleven healthy male volunteers [age (mean ± SD), 22.3 ± 4.0 years; range, 20–34 years] were selected to participate. Before experiments, we assessed their MI ability using the revised Vividness of Movement Imagery Questionnaire (VMIQ-2). The VMIQ-2 consists of 12 items. Three different imagery perspectives, including internal visual imagery, external visual imagery, and kinesthetic imagery, are required in each item. Subjects rate the vividness of their imagery using a 5-point Likert scale, with 1 as perfectly clear and vivid and 5 as no image at all, you only know that you are “thinking” of the skill. In this study, we asked the subjects to perform kinesthetic imagery (see the “Motor Imagery Task” section). Thus, only the kinesthetic imagery score was employed. A score of 36 or less on kinesthetic imagery was required to be included in this study. The mean score was 24.7 ± 6.5 and no one had a score higher than 36. Thus, all subjects who participate in this study had sufficient ability for kinesthetic imagery. All of our previous studies adopted a non-dominant hand movement for the MI task. All subjects were determined to be right-handed using the Edinburgh Handedness Inventory (Oldfield, 1971). Written informed consent was obtained before participating in this study. The study was approved by the Research Ethics Committee at Kansai University of Health Sciences (approval number: 16–43) and was conducted in accordance with the Declaration of Helsinki.

### Experimental Procedures

#### Motor Execution Task

**FIGURE 1:**
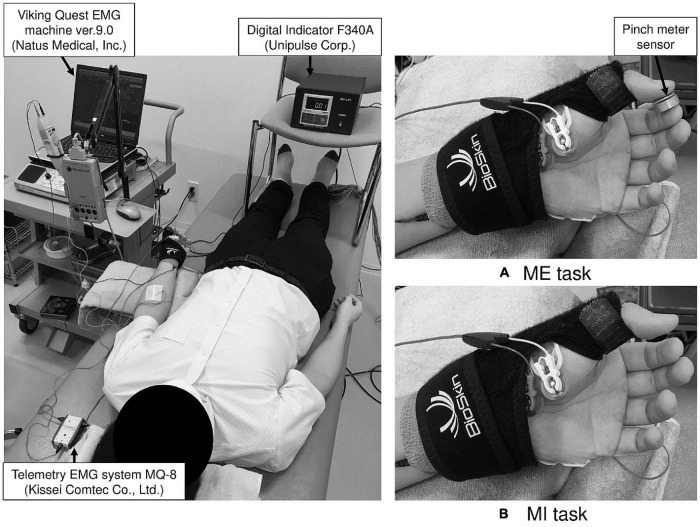
The experimental setup for the motor execution (ME) and motor imagery (MI) task. During examination, the left thumb and index finger were fixed in opposition position using a splint to maintain the same position in the ME **(A)** and MI **(B)** tasks.

Subjects assumed a supine position on a bed and focused on the display of the pinch meter (Digital Indicator F340A; Unipulse Corp., Tokyo, Japan) throughout the experiment. The left forearm was fully supinated. The left thumb and index finger were fixed in opposition position with a Bio Skin™ Thumb Spica splint (Bio Skin, Inc., Medford, OR, United States), and the other fingers were relaxed (Figure 1). We confirmed that the subjects’ position did not change during the examination. Prior to F-wave recording for the MI task and to confirm EMG activity of finger muscles not involved in actual movement (enslaving), we measured the muscle activity of the left ADM during isometric opposition pinch movement by the left thumb and index finger at 50% MVC. To detect the maximal pinch force, subjects pressed the pinch meter sensor between the left thumb and index finger with maximal effort for 5 s. The pinch force value, which is expressed as kilogram-force (kgf), was measured using EMG recording software (Vital Recorder 2; Kissei Comtec Co., Ltd., Matsumoto, Japan) and was analyzed using a multiple biological information analysis system (BIMUTAS-video; Kissei Comtec Co., Ltd., Matsumoto, Japan). Maximal pinch force was calculated by averaging the maximal pinch force exerted in three trials. Then, subjects were informed of their target pinch force (i.e., the pinch force at 50% MVC). To determine the baseline level of muscle activity, after a 5-min break, EMG was recorded during rest for 10 s. For the ME condition, subjects exerted the pinch force at 50% for 10 s with visual feedback. Specifically, they adjusted to the target pinch force while viewing the pinch force value numerically displayed in real time on the pinch meter. To confirm subjects could adjust pinch force at their target value, we compared exerted pinch force during the ME condition with the target pinch force. Simultaneously, muscle activity while exerting the pinch force at 50% was recorded from the left ADM. Specific instructions on middle, ring, and little finger movements were not provided for the ME task, and subjects were instructed to perform isometric opposition pinch movement by only the thumb and index finger.

#### Motor Imagery Task

To study whether MI of isometric thenar muscle activity affects spinal motor neuron excitability corresponding to ADM muscle, F-wave recordings were conducted under two experimental conditions termed Rest and MI. Because slight muscle contraction can alter spinal motor neuron excitability (Hara et al., 2010), background EMG recording was performed concurrently with F-wave recording. In the Rest condition, F-waves and background EMG were recorded from the left ADM during relaxation for 1 min to determine the baseline level of spinal motor neuron excitability and EMG activity. Before performing MI, the subjects were instructed to learn left thenar muscle contraction by exerting pinch force at 50% (i.e., left thenar muscle contraction at 50% MVC) for 1 min. As mentioned above, the target pinch force was adjusted using visual feedback. Then, in the MI condition, the subjects imagined left thenar muscle contraction at 50% MVC for 1 min. The F-wave and background EMG were recorded from the left ADM during MI. Additionally, to confirm subjects could perform MI of isometric thenar muscle activity at 50% MVC without any muscle contraction, background EMG was recorded from the left APB, which enables thumb opposition movement and is innervated by the median nerve alone (Skoff, 1998), during the Rest and MI conditions. Attention may affect the spinal motor neuron excitability (Bathien and Morin, 1972). Thus, as in the ME task, specific instructions on middle, ring, and little finger movements were not provided for the MI task.

#### Recording Apparatus and Condition for F-Waves

**FIGURE 2:**
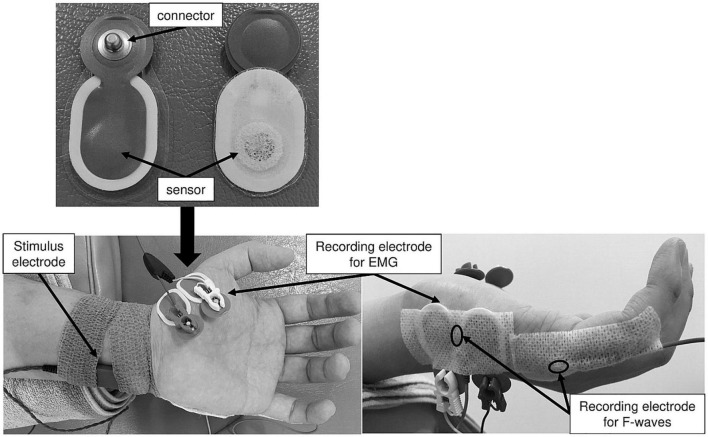
The recording electrode arrangement for the ME and MI task. These figures show placement of stimulus and recording electrodes for the ME and MI tasks.

A Viking Quest EMG machine version 9.0 (Natus Medical, Inc., Pleasanton, CA, United States) was used for the F-wave recording. A pair of silver EEG cup electrodes (10-mm diameter; Natus Medical, Inc., Pleasanton, CA, United States) was attached over the left ADM muscle eminence and the base of the fifth dorsal metacarpal bone (Figure 2). To maintain skin impedance < 5 kΩ, the skin was cleaned with an abrasive gel (Nuprep^®^ Skin Prep Gel; Weaver and Company, Inc., Aurora, CO, United States). F-waves were evoked from the left ADM muscle by delivering supramaximal electrical stimulation to the left ulnar nerve at the wrist. Supramaximal stimulus intensity was assigned to be 20% higher than the maximal stimulus intensity that could elicit the largest amplitude of M-waves. Thirty supramaximal electrical stimuli were delivered at a duration of 0.2 ms and frequency of 0.5 Hz (i.e., every 2 s) in each experimental condition. The signal-to-noise ratio was 110 db. Amplifier gains of 5 mV per division for M-waves and 200 μV per division for F-waves were used. The bandwidth filter ranged from 20 Hz to 3 kHz. All F-wave recordings were conducted by the same investigator in the same laboratory and under the same conditions (room temperature set at 25°C).

#### Recording Apparatus and Condition for Electromyography Activity

Surface EMG recording was conducted using a telemetry EMG system (MQ-8; Kissei Comtec Co., Ltd., Matsumoto, Japan) and EMG recording software (Vital Recorder 2; Kissei Comtec Co., Ltd., Matsumoto, Japan). A pair of disposable Ag/AgCl electrodes (Blue Sensor N-00-S; Ambu A/S, Ballerup, Denmark) was attached over the left APB and ADM muscles with an interelectrode distance of 15 mm (Figure 2). The EMG data recorded from the left APB and ADM muscles were filtered with a low-pass frequency of 10 Hz and high-pass frequency of 1 kHz and digitized at a sampling frequency of 2 kHz.

### Data Analysis

First, to study the dynamics of spinal motor neuron excitability corresponding to the ADM muscle using F-waves, antidromic impulses from all motor neurons should reach the spinal anterior horn cells. Considering bidirectional conduction, the characteristic of nerve impulse conduction, to confirm supramaximal electrical stimuli could excite all α-motor neurons, the M-wave amplitudes during two experimental conditions were compared. All F-wave data were analyzed with respect to F-wave persistence and F-wave amplitude. In this study, the minimum peak-to-peak amplitude of F-waves was 20 μV (Fisher, 2002; Hara et al., 2010). F-wave persistence is defined as the number of detected F-wave responses divided by the number of electrical stimulations and is expressed as a percentage (%). The F/M amplitude ratio, which is defined as the mean amplitude of all detected F-wave responses divided by the M-wave amplitude and is expressed as a percentage (%), was calculated to normalize F-wave amplitude. F-wave persistence and the F/M amplitude ratio are indices of spinal motor neuron excitability. Specifically, F-wave persistence reflects the number of backfiring spinal anterior horn cells (Mesrati and Vecchierini, 2004; Fisher, 2007), and the F/M amplitude ratio reflects the number, size, and synchronization of backfiring spinal anterior horn cells (Peioglou-Harmoussi et al., 1985; Mesrati and Vecchierini, 2004). Additionally, the latency between F-waves and M-wave was calculated. The latency is defined as the mean latency from the time of stimulation to onset of a measurable M-wave and F-waves and is expressed as a millisecond (ms).

Raw EMG signals in each condition were filtered with a low-pass frequency of 10 Hz and high-pass frequency of 1 kHz and then converted from analog to digital at a sampling frequency of 2 kHz. For EMG activity from the left ADM muscle while exerting pinch force at 50%, the root mean square (RMS) values were calculated from raw EMG signals during the Rest and ME conditions. For background EMG recording, the RMS values were calculated from raw EMG signals from the left APB muscle during the Rest and MI conditions. Also, for background EMG of the left ADM muscle during the Rest and MI conditions, to avoid the influence of the stimulus artifact for eliciting the F-waves from the left ADM muscle, analysis areas were selected from interelectrical stimulation. The duration of each area was 1 s, and thus, the duration of analysis was 30 s.

### Statistical Analysis

IBM SPSS statistics version 26 (IBM Corp., Armonk, NY, United States) was used for the statistical analysis. First, the normality of all measured data was rejected by the Shapiro–Wilk test. Thus, a non-parametric method was used in this study. The concrete statistical methods for each experimental procedure were as follows: in the statistical analyses for the ME task, exerted pinch force value during the ME condition was compared with the target pinch force value. The RMS values obtained from the two experimental conditions (Rest and ME) were compared. In the statistical analyses for the MI task, the M-wave amplitude and RMS values obtained from the two experimental conditions (Rest and MI) were compared initially, respectively. Subsequently, F-wave persistence, amplitudes, the F/M amplitude ratios, and the latencies between F-waves and M-wave obtained from the two experimental conditions (Rest and MI) were compared, respectively. In this study, Wilcoxon signed-rank test was used for intragroup comparison. Furthermore, the statistical power (1−β error probability) and an effect size [Pearson’s correlation coefficient (*r*)] were calculated in all significant data by a *post hoc* power analysis with G*power version 3.1.9.4 (Faul et al., 2007). The significance level was set at α = 0.05.

## Results

### Pinch Force and Electromyography Activity During the Motor Execution Task

No significant difference was observed between the pinch force value during the ME condition and the target pinch force value (Wilcoxon signed-rank test, *z* = −0.133, *p* = 0.894, Table 1).

**TABLE 1:** The comparison for pinch force between the motor execution (ME) condition and the target pinch force.

	Target pinch force	ME condition	Significance
Pinch force (kgf)	1.314 ± 0.380	1.323 ± 0.395	n.s.

*n.s., not significant.*

**FIGURE 3:**
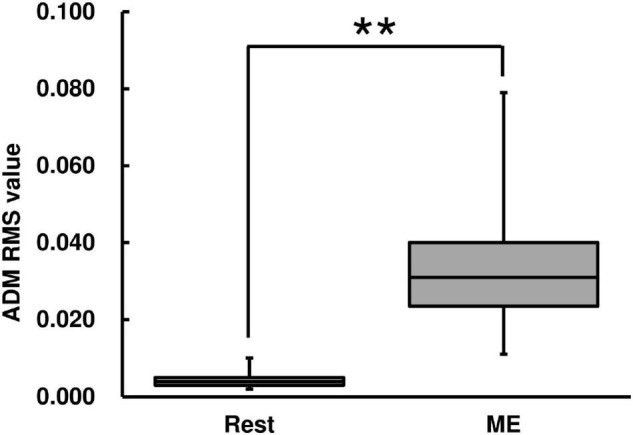
Root mean square (RMS) value of abductor digiti minimi (ADM) muscle for the ME task. RMS values are represented using a box and whisker plot, with the median shown as the middle horizontal line, the upper and lower quartiles displayed as boxes, and the maximum and minimum represented as whiskers. The vertical axis shows the RMS value, and the horizontal axis shows the specific condition (Rest and ME). **p < 0.01.

The RMS value of the left ADM muscle during the ME condition was higher than that during the Rest condition [Wilcoxon signed-rank test, *z* = −2.93, *p* < 0.01, *r* = 0.625, (1−β) ≈ 100%, Figure 3].

### M-Waves, F-Waves, and Background Electromyography During the Motor Imagery Task

There was no difference in the M-wave amplitude between the Rest and MI conditions (Wilcoxon signed-rank test, *z* = −0.891, *p* = 0.373, Table 2).

**TABLE 2:** The M-wave and F-waves and background electromyography (EMG) for the motor imagery (MI) task.

	Rest condition	MI condition	Significance
M-wave amplitude (mV)	16.9 ± 3.62	16.9 ± 3.66	n.s.
F-wave amplitude (mV)	0.151 ± 0.061	0.254 ± 0.095	**p < 0.01
Latency between F-wave and M-wave (ms)	25.7 ± 2.60	25.5 ± 2.62	n.s.
RMS value (ADM)	0.004 ± 0.002	0.005 ± 0.002	n.s.
RMS value (APB)	0.005 ± 0.004	0.005 ± 0.003	n.s.

*MI, motor imagery; ADM, abductor digiti minimi muscle; APB, abductor pollicis brevis muscle; n.s., not significant.*

**FIGURE 4:**
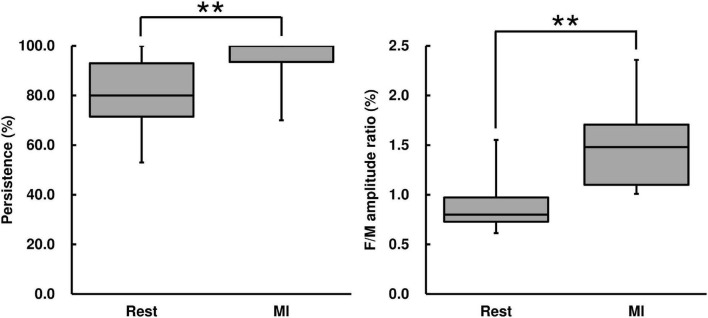
F-wave persistence and the F/M amplitude ratio for the MI task. F-wave persistence and the F/M amplitude ratio are represented using a box and whisker plot, with the median shown as the middle horizontal line, the upper and lower quartiles indicated as boxes, and the maximum and minimum represented as whiskers. The vertical axis shows F-wave persistence (%) and the F/M amplitude ratio (%), and the horizontal axis shows the specific trial (Rest and MI). **p < 0.01.

F-wave persistence during the MI condition was higher than that during the Rest condition [Wilcoxon signed-rank test, *z* = −2.67, *p* < 0.01, *r* = 0.569, (1−β) = 99.5%, Figure 4]. F-wave amplitude during the MI condition was higher than that during the Rest condition [Wilcoxon signed-rank test, *z* = −2.93, *p* < 0.01, *r* = 0.625, (1−β) ≈ 100%, Table 2]. The F/M amplitude ratio during the MI condition was also higher than that during the Rest condition [Wilcoxon signed-rank test, *z* = −2.93, *p* < 0.01, *r* = 0.625, (1−β) ≈ 100%, Figure 4]. The latency between F-waves and M-wave during the MI condition did not differ from that during the Rest condition (Wilcoxon signed-rank test, *z* = −0.939, *p* = 0.348, Table 2).

There was no difference in the RMS value measured from the left APB muscle between the Rest and MI conditions (Wilcoxon signed-rank test, *z* = −1.14, *p* = 0.256, Table 2). Also, there was no difference in the RMS value measured from the left ADM muscle between the Rest and MI conditions (Wilcoxon signed-rank test, *z* = −0.520, *p* = 0.603, Table 2).

A *post hoc* power analysis showed that all significant results reached a power > 90% based on sample size (*n* = 11) and achieved a sufficient effect size and statistical significance level (α = 0.05). The result of power analysis indicates that this study has sufficient power to detect statistical significance (Cohen, 1992).

## Discussion

In the ME task, there was no difference between exerted pinch force value during the ME condition and the target pinch force value, and thus, subjects were able to exert pinch force at their target contraction strength. Furthermore, in the MI task, there were no differences in both the M-wave amplitude evoked from the left ADM and RMS values obtained from the left ADM and APB muscles between the Rest and MI conditions. These results indicate that changes in whole spinal motor neuron excitability were evaluated in this study and that the subjects could perform the MI task without any muscle contraction. As described above, slight muscle contraction can easily alter spinal motor neuron excitability (Hara et al., 2010). Thus, alterations of the F-wave parameters are considered to be due to the effect of MI only, and the influence of muscle contraction accompanying MI could be excluded.

For the ME task, we first investigated whether muscle activity of the uninstructed little finger occurs during an isometric opposition pinch movement by the thumb and index finger. The RMS value of the left ADM muscle was significantly increased during ME, and thus, unintentional finger force production (enslaving) effect was confirmed in this study. This result agrees with a number of previous findings (Kilbreath and Gandevia, 1994; Zatsiorsky et al., 1998, 2000; Yu et al., 2010). There are biomechanical and neurophysiological constraints associated with the enslaving effect (Zatsiorsky et al., 1998, 2000; Schieber and Santello, 2004). For biomechanical constraints, the anatomical design (i.e., multitendon and multidigit) of the extrinsic flexors and passive connective tissue links between adjacent fingers contribute to the enslaving effect (Leijnse et al., 1993; Kilbreath and Gandevia, 1994). However, the thumb does not share multidigit muscles with other fingers and its passive mechanical links to other fingers are very small (Olafsdottir et al., 2005; Yu et al., 2007, 2010). Moreover, the unintentional force production caused by passive mechanical interactions not likely to require any muscle activity. Thus, the observed left ADM muscle activity during an isometric opposition pinch movement by the left thumb and index finger may be due to neurophysiological constraints rather than biomechanical constraints. Previous neurophysiological studies in the primary motor cortex have shown extensive overlap of territories activated during the movements of different digits (Schieber, 1991; Schieber and Hibbard, 1993; Schieber and Santello, 2004). Furthermore, output projections from single neurons of the primary motor cortex diverge to innervate the motoneuron pool of more than one muscle (Shinoda et al., 1979, 1981). Indeed, unintentional force production of the little finger was confirmed during thumb or index finger movement at MVC (Zatsiorsky et al., 2000; Yu et al., 2010).

Subsequently, for the MI task, we investigated whether the enslaving effect occurs in spinal motor neuron excitability corresponding to the left ADM (uninstructed) muscle during MI of isometric left thenar muscle activity. Both F-wave persistence and the F/M amplitude ratio measured from the left ADM muscle were significantly increased during MI. As described in the Introduction, MI is the mental representation of movement without any overt motor output. Thus, and also the ME task, the present F-wave and background EMG results suggest that enhancement of spinal motor neuron excitability corresponding to an uninstructed finger muscle during MI of instructed finger muscle contraction may be due to neurophysiological factors, not biomechanical factors. Concerning the enslaving effect during MI, previous studies using TMS demonstrated that MI increased the corticospinal excitability of the prime mover muscle associated with mentally simulated finger movement (Rossini et al., 1999; Facchini et al., 2002; Fourkas et al., 2006). Results of those studies indicate that there is a movement-specific modulation of corticospinal excitability during MI. A similar tendency was observed in spinal motor neuron excitability (Rossini et al., 1999; Facchini et al., 2002). Enhancement of corticospinal excitability was confirmed in both instructed and uninstructed muscles during MI (Li et al., 2004; Stinear and Byblow, 2004). These previous results indicate that the enslaving effect on corticospinal excitability occurs during MI. The methodological differences among the previous studies may explain the contradictions in results. If MI shares a neural foundation with actual movement, MI may mentally simulate muscle activities accompanied with actual movement. However, previous studies did not assess the corticospinal excitability corresponding to uninstructed fingers during actual movements by instructed fingers (Rossini et al., 1999; Fourkas et al., 2006). Thus, the results of these previous studies may not rule out the possibility of an enslaving effect during MI, and also during actual movement. Previous studies indicate that MI specifically affects effectors that would be involved in actual movement (Facchini et al., 2002; Li et al., 2004). Furthermore, it should be noted that there is similarity of intracortical inhibition to muscles not involved in ME between MI and ME (Stinear and Byblow, 2004). Such an inhibitive modulation of the motor cortex during MI was also observed by Liepert and Neveling (2009). Interestingly, Aoyama et al. (2019a) reported that corticospinal excitability corresponding to the FDI muscle was suppressed during combined MI and action observation of index finger adduction movement; however, spinal motor neuron excitability was significantly increased. This result indicates that cortical-mediated inhibition may involve inhibitory changes in corticospinal excitability corresponding to the FDI as antagonist muscle to execute index finger movement selectively, whereas spinal involvement does not contribute. Additionally, when subjects imagined index finger abduction movement, as selectively as possible, while observing a movie of actual movement, amplitude of MEPs obtained from the ADM muscle, which coactivates with the FDI muscle during hand-opening movement, remained unchanged, while spinal reflex excitability of the ADM muscle was increased (Aoyama et al., 2019b). This contradictory result suggests that cortical inhibitory mechanisms might have been recruited to counterbalance the increased spinal excitability. Aoyama et al. (2019a,b) tried to clarify the neurophysiological mechanisms involved in selective finger movement at cortical and spinal level using combined MI and action observation, whereas this study aimed to investigate changes in spinal motor neuron excitability corresponding to unintended finger muscle during MI of finger movement by the prime mover muscle. In other words, subjects in this study were not required to perform thumb finger movement as selectively as possible and were given no specific instructions on finger movements except the thumb. In addition, Li et al. (2004) investigated the enslaving effect by comparing TMS-induced forces from the four fingers and not the thumb during rest and MI condition. MI of pressing movement by the index finger at MVC increased TMS-induced force production by unintentional fingers, specifically the middle, ring, and little fingers. However, previous studies performed by Li et al. (2004) did not assess spinal motor neuron excitability. Consequently, although there are differences in aims and methodologies among the studies, these previous findings indicate that MI may simulate the facilitative and inhibitive neural activities that occur during actual movement. In this study, spinal motor neuron excitability corresponding to the ADM muscle, which is an unintended finger muscle, was significantly increased during MI of isometric thenar muscle activity at 50% MVC without any muscle contraction of both the prime mover (APB) and unintended muscle (ADM). Unfortunately, because the artifact by muscle activity would be mixed in F-waves recorded from the ADM muscle during the ME condition, we could not directly compare spinal motor neuron excitability between the MI and ME conditions, but we confirmed the muscle activity of the ADM muscle during actual isometric pinching movement by thumb and index finger. Thus, this is the first study of neurophysiological factors of the enslaving effect at the spinal level by demonstrating a significant increase in spinal motor neuron excitability corresponding to unintended muscle during MI of thenar muscle activity.

As a first limitation, we recruited only young men in this study. MI ability (i.e., how vividly subjects can perform MI) is decreased in elderly people compared with younger adults (Saimpont et al., 2013). Indeed, the excitability of central nervous system was influenced by MI ability (Lorey et al., 2011; Williams et al., 2012). In addition, gender affects MI ability. The ability to mentally rotate objects is greater in men than women (Campos, 2014). Thus, we cannot rule out the influence of sampling bias on the effect of MI on spinal motor neuron excitability. As a second limitation, because we only assessed spinal motor neuron excitability using F-waves, the behavior of the central nervous system for the enslaving effect during the MI task adopted in this study is unclear. Finally, spinal motor neuron excitability corresponding to the APB muscle, the prime mover of a pinching movement, during the MI task was not measured. Thus, further research is required to resolve these limitations. However, our laboratory has provided strong evidence that MI of isometric thenar muscle activity at 50% MVC increases spinal motor neuron excitability corresponding to the APB muscle (Suzuki et al., 2013; Bunno et al., 2014, 2015; Bunno, 2018, 2019).

## Conclusion

In this study, subjects imagined isometric thenar muscle activity at 50% MVC. Furthermore, a specific instruction on little finger movement was not given for the MI task. Nevertheless, spinal motor neuron excitability corresponding to the ADM muscle was significantly increased during MI without any overt muscle activity. Thus, the present results suggest that MI may modulate spinal motor neuron excitability similar to that observed in actual movement. To conclude, MI may reproduce the pattern of muscle activities involved in ME.

## Data Availability Statement

The raw data supporting the conclusions of this article will be made available by the authors, without undue reservation.

## Ethics Statement

The studies involving human participants were reviewed and approved by the Research Ethics Committee at Kansai University of Health Sciences. The patients/participants provided their written informed consent to participate in this study.

## Author Contributions

YB: conceptualization, methodology, investigation, data curation, formal analysis, visualization, and writing-original draft. TS: validation, writing-review and editing, supervision, and project administration. Both authors contributed to the article and approved the submitted version.

## Conflict of Interest

The authors declare that the research was conducted in the absence of any commercial or financial relationships that could be construed as a potential conflict of interest.

## Publisher’s Note

All claims expressed in this article are solely those of the authors and do not necessarily represent those of their affiliated organizations, or those of the publisher, the editors and the reviewers. Any product that may be evaluated in this article, or claim that may be made by its manufacturer, is not guaranteed or endorsed by the publisher.
